# Dentogingival Smile Analysis of Young Adults of Al Qassim Province, Saudi Arabia: A Cross-Sectional Study

**DOI:** 10.1155/2020/8855681

**Published:** 2020-11-10

**Authors:** Minu P. Mohan, Rahaf A. AlOlayan, Mohammad A. AlSweed

**Affiliations:** ^1^Assistant Professor, Department of Prosthetic Dental Sciences, College of Dentistry, Al Qassim University, Buraydah 51451, Saudi Arabia; ^2^Dental Intern, College of Dentistry, Al Qassim University, Buraydah 56219, Saudi Arabia; ^3^General Practitioner, Private Practice, Buraydah 52353, Saudi Arabia

## Abstract

**Objectives:**

The present study aims to analyse the smiling components among young adults within Al Qassim region by evaluating typical smile parameters. *Methodology*. A cross-sectional study was conducted among 324 female and male participants between 18 and 35 years, within Al Qassim Province of Saudi Arabia. The dentogingival macro-aesthetic elements of the smile, the parallelism between the incisal curve and lower lip line, midline, and buccal corridors were determined by using Canon Rebel t7i (Canon, Melville, NY, USA) and evaluated by photo editing software Adobe Photoshop CC2019. The data were analysed using a chi-square test and Spearman's correlation test for nonparametric data.

**Results:**

62.5% of young adults had no buccal corridors. The nonparallel smile was found in 53.2% of young adults. A high smile line was observed in 33% of participants. 59.3% of young adults lacked a coinciding dental and facial midline. There was a statistically significant difference in the parallelism of the incisal curve and lower lip line as well as in the position of the upper lip line across genders (*P* < 0.05).

**Conclusion:**

Understanding macro-aesthetic elements of teeth and their interrelation with the surrounding oral structures can be a guide in creating natural and aesthetically pleasing restorative treatment.

## 1. Introduction

Smiling is one of the most critical facial expressions and is known as a nonverbal parameter of correspondence [1]. Cosmetic dentistry plays a significant role in improving the smile of a patient. A systematic and comprehensive dentofacial analysis must be performed before commencing aesthetic treatment [2]. Creating an aesthetically pleasing smile requires the integration of facial and dental parameters. Dental and facial aesthetics can be defined in terms of macro- and microelements [3]. Macro-aesthetic components include interrelationships between the face, lips, gingiva, and teeth. The aesthetics of an individual tooth and its color and form comprise micro-aesthetics [3]. Analysis of smile integrates facial, dentofacial, and dental aesthetics, encompassing the macro- and microelements [3].

Several studies involving different populations have provided evidence that aesthetic components for different sexes, races, and ages are not entirely the same [4–6]. However, the number of studies conducted to assess smile parameters within the Saudi population is minimal [6, 7]. Additionally, results of these studies were variable and inconclusive. Furthermore, none of these studies were conducted within Al Qassim region to assess and analyse different smile parameters [6, 7].

Appreciation of what society considers acceptable and aesthetically pleasing is crucial for a successful outcome of prosthetic and restorative treatment [8, 9]. The characteristic feature of a smile, described by several researchers, has served as a guideline for restoration enhancement for the anterior component of the dentition [8, 10–12]. Smile analysis—obtaining averages of the smile's characteristic features among various populations—provides an understanding of these features' general pattern of occurrence [12].

Tjan et al. formulated a standard of normalcy in an aesthetic smile relative to the smiling type: high, average, and low; parallelism of the maxillary incisal curve with the lower lip; and the number of teeth seen when smiling [1].

The buccal corridor is a significant aesthetic parameter [13–16]. It is defined as the negative spaces between the facial surfaces of the posterior teeth and the commissure of the mouth when the patient smiles. The buccal corridor space's width influences smile attractiveness in different facial types [15].

The coincidence of the dental midline and the facial midline is a controversial subject in aesthetics, and many researchers suggest that it is more important for the facial midline and the maxillary dental midline to coincide rather than the facial and mandibular midlines. The dominant visibility of the maxillary teeth when smiling is a primary reason behind this argument [17–19]. The coincidence of facial, maxillary, and mandibular midlines is favourable but not mandatory [17]. In recent years, attempts to include smile analysis and smile aspects into treatment planning have become the key to prosthodontic treatment success [12, 20, 21].

The null hypothesis of this study is that there would be no difference in the distribution and analysis of smile parameters among young Saudis within Al Qassim Province. A cross-sectional study was conducted to assess the dental-gingival macro-aesthetic elements of a smile, the position of the upper lip line, the parallelism of the incisal line and lower lip line, location of the arch midline, and presence or absence of the buccal corridor among participants. Findings may be used as a guide for advanced smile designing and restorative procedures for this population.

## 2. Materials and Methods

The study was approved by the ethical committee at Al Qassim University, KSA (SRPSSC), approval number: F-2018-3018, registered on 29 September 2018 following the ethical principles of research involving human subjects.

The cross-sectional study was conducted within Buraydah, Unizah, and Arras regions of Al Qassim Province. A pilot study was conducted previously to calculate the sample size. The total sample size contained 324 participants, comprising 162 males and 162 females, who reported to 10 dental clinics across Al Qassim Province. Informed consent was obtained from all 324 participants. The target study population included Saudi young adults between 18 and 35 years. Non-Saudi participants were excluded from the study. The present study also excluded participants who had prosthodontic crowns or orthodontically modified dentition. In fact, both prosthodontic frameworks and orthodontic appliances can alter aesthetic appearances [22, 23]. Additionally, participants who had bleached dentition, dental fluorosis, and hypoplasia were excluded from the study.

### 2.1. Photography

A standardized photograph (frontal view) was taken of each participant's full social smile (portrait) by a trained photographer, using Canon Rebel t7i (Canon, Melville, NY, USA). The settings were standardized to 1/60 second shutter speed, F5.6, and ISO 400. A macroring flash (Yongnuo YN-14EX) was attached to the lens for standardized lighting. Canon tripod was adjusted individually according to the participant's height. The participant was seated at a 90° head position on the dental chair. The photographs were captured 25 cm away from the participant's nose [24].

### 2.2. Smile Analysis

To assess the dentogingival components of the smile, Adobe Photoshop CC2019 (Adobe Inc, CA, USA) was used. The position of the upper lip line was located by drawing a line following the upper lip contour and gingival zenith of the upper anterior teeth (teeth included from #13 to #23). The upper lip line was considered high when the observed distance between the gingival zenith and upper lip line was 2-3 mm, medium when the display of 75–100% clinical crown height was seen, and low when the upper lip covered 25% or more of the labial surface of the upper anterior teeth [7].

**Figure 1:**
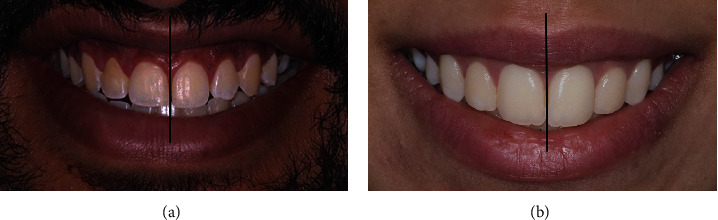
(a) Male subject showing no buccal corridor and no midline shift. (b) Female subject showing no buccal corridor and no midline shift.

**Figure 2:**
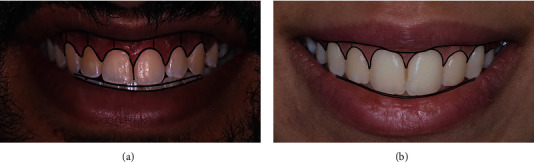
(a) Male subject showing parallel smile and high lip line. (b) Female subject showing parallel smile and medium lip line.

The parallelism between the incisal curve and the lower lip line was determined by drawing two curves. The facial midline was located by drawing a line through the base of the philtrum. The dental midline was located using a line drawn through the central incisors. The deviation of the dental midline from the facial midline was recorded independently in pixels using a ruler tool (Adobe Photoshop CC2019, Adobe Inc., CA, USA) and was then converted into millimeters. The presence or absence of the buccal corridor between the posterior teeth and the commissures of the mouth when smiling was evaluated using a magnetic lasso tool (Adobe Photoshop CC2019, Adobe Inc., CA, USA), following the buccal corridor area (Figures 1 and 2).

### 2.3. Statistics

SPSS (IBM SPSS V25, IBM Corporation, Chicago, IL, USA) was used for data analysis. A descriptive analysis was done for all aforementioned variables: the position of the upper lip, incisal line or parallelism, position of the midline, amount of shift, and presence or absence of the buccal corridor.

Chi-square, Mann–Whitney *U*, and independent *t*-tests were used to investigate if there was a significant difference between male and female smiles. For all statistical testing, *P* = 0.05.

## 3. Results

**Figure 3:**
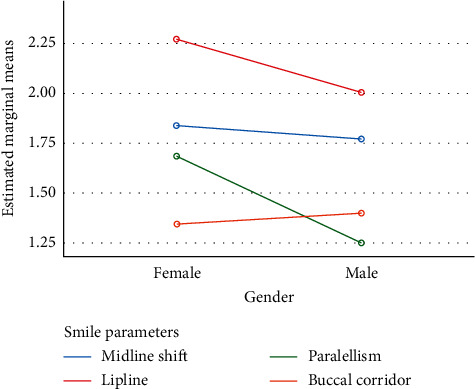
Plot of the mean score for smile parameters and gender.

The obtained data were arranged systematically, using SPSS V25. The collected information was transferred from a table, created with Excel 2016. Data from all 324 participants (162 females and 162 males) were analysed. The distribution of smile parameters among the participants is explained in Table 1. The plot of the mean score for each combination of groups of “gender” and “smile parameters” is presented using a line graph (Figure 3).

### 3.1. Smile Line

The distribution of female participants' smile line parameters was as follows: 51.2% with a medium smile line, 24.7% with a high smile line, and 24.1% with a low smile line. For male participants, 41.4% had a high smile line, 29.6% had a medium smile line, and 29% had a low smile line. There was a statistical difference between females and males in smile line distribution when using the Mann–Whitney *U* test for nonparametric data (Table 2).

### 3.2. Buccal Corridor

Buccal corridors were absent in 65.4% of females and 59.9% of males. A chi-square test was applied to test the difference between males and females in the presence or absence of a buccal corridor, and no statistical difference was found (Table 2).

### 3.3. Parallelism

The parallelism between the incisal curve and the lower lip line was classified as parallel and nonparallel. Parallelism was found in 68.5% of females and 24.7% of males, while 31.5% of females and 75.3% of males had nonparallel smiles. The chi-square test showed that the difference between males and females for the parameter parallelism was statistically significant (Table 2).

### 3.4. Midline Shift

Midline shift was divided into two categories: 0-1 mm and ≥2 mm midline shifts (Table 1). 56.2% of males and 62.3% of females had their dental midline shifted 2 mm or above. A chi-square test was applied to detect the difference between females and males in the distribution of midline shift, which showed no statistically significant difference (Table 2).

### 3.5. Association between Smile Parameters

Spearman's rho correlation test for nonparametric data was applied to investigate the association between the four different variables. Results show a statistically significant positive weak relation between the midline shift and the buccal corridor (“*ρ*” (rho) = 0.139, *P* < 0.05). There was no other statistically significant association between smile parameters (Table 3).

## 4. Discussion

Currently, few criteria are used for smile analyses of young individuals [6, 7, 10, 12, 24, 25]. The purpose of this study was to evaluate and analyse dentogingival macro-aesthetic elements of the smile of young adults within Al Qassim Province. Although limited research has described the smile components of young adults in Saudi Arabia, research has yet to be conducted within Al Qassim region [6, 7].

Tjan et al. discussed three types of smile lines: a high smile line, which reveals the full cervical-incisal length of the anterior maxillary teeth with a contiguous band of the gingiva; an average smile line that reveals 75%–100% of the upper anterior teeth; and a low smile line that reveals less than 75% of the upper anterior teeth [1]. In the current study, the average smile line was observed to be the most prevalent among participants (seen in 40.4% of the sample). Other studies have also reported similar findings [1, 12]. For instance, the smile line was studied by Alqarni et al. within Asser region. These authors found that the average smile line was more commonly seen, compared to a high or low smile line [7]. Nold et al. also investigated the smile line position among Turkish populations. They concluded that a high smile line was a common feature seen in females, while a medium smile line was mostly seen in males [26]. In the current study, we found that a medium smile line was more prevalent in females and a high smile line was more prevalent in males (51.2% and 41.4%, respectively).

The influence of buccal corridors on smile aesthetics was studied by Albwardi et al. They concluded that excessive teeth display with 2% buccal corridors was considered to be interpreted as the least attractive smile. However, a medium broad smile with 10% buccal corridor was considered as the most attractive [27]. Liang et al. also studied buccal corridors in 188 Chinese females and males and found that the buccal corridor was present in 69% and 51.1%, respectively [25]. Interestingly, in the present study, the buccal corridor was absent in 65.4% of females and 59.9% of males.

The incisal curve is known to be ideal when the convex curve follows the concavity of the lower lip when smiling [28]. The phrase consonant used to describe this parallel relationship, a nonconsonant or flat, smile arc can be seen when the maxillary incisal curve is flatter than the curve of the lower lip when smiling [29]. Soares et al. concluded that a straight and convex incisal curve was more common than a reverse incisal curve [28]. Moreover, Al-Johany et al. studied 50 participants and found that 78% had an incisal curve that was parallel with the lower lip [30]. With that said, we found that only 24.7% of male participants had a parallel smile, with a higher percentage in females (68.5%). This difference in the parallel smile between male and female participants was statistically significant. These results were consistent with Nold et al. who concluded that there was a statistical difference between genders in the distribution of the incisal curve, with the parallel smile more commonly seen in females rather than males [26].

The midline is a crucial vertical reference line [31]. Miller et al. studied midline discrepancy and found that, in 70% of cases, the dental midline coincides with the facial midline [19]. Moreover, these authors stated that minimal deviations in the midline do not affect overall aesthetics. Al-Balkhi and Zahrani found that the prevalence of the midline shift was 30.7% [31]. However, we found that 51.6% of males had more than or equal to a 2 mm midline shift, and 52% of female participants had more than or equal to a 2 mm midline shift. We assessed the dental midline by using central incisors as a reference point, while the facial midline was assessed by using the philtrum as a reference point. This approach is considered a valid method for evaluating the midline shift [19].

A limitation of the present study is the limited sample size taken from a single province in Saudi Arabia. The inclusion of a larger representative sample from multiple regions in Saudi Arabia would help investigate whether there is a statistical difference between different regions in terms of the distribution in smile parameters. Further facial reference points were needed for facial midline determination to precisely measure the amount of midline shift. However, cultural principles and religious considerations precluded taking any wider photographs, especially regarding female subjects. Incorporation of additional smile parameters in future research, such as the geometry, proportion, and shade of the anterior teeth, can be beneficial.

## 5. Conclusion

The present study aimed to analyse the smiling components among young adults within Al Qassim region by evaluating typical smile parameters. We found that the parallelism of the smile arc and lower lip line was statistically higher in females than males in Al Qassim region. Additionally, there was a statistically significant difference between genders in the position of the lip line. These results underline the importance of smile parameters when restoring a patient's intramural harmony. Moreover, in order to obtain adequate results in oral rehabilitation, it is necessary to take an individual approach to assess each patient while taking into account their own expectations and preferences.

## Figures and Tables

**Table 1:** Frequency distribution table of different smile parameters.

		Frequency	%
Gender	Female	162	50.0
	Male	162	50.0

Midline shift	0-1 mm	132	40.7
	≥2 mm	192	59.3

Lip line	Low	86	26.5
	High	107	33.0
	Medium	131	40.4

Parallelism between lower lip and smile line	No	173	53.4
	Yes	151	46.6

Buccal corridor	Absent	203	62.7
	Present	121	37.3

**Table 2:** Chi-square test for the association between gender and different smile parameters.

			Female	Male	Pearson chi-square	*P* value^*∗*^
Midline shift	0	Count	18	20	1.892	0.756
		% within gender	11.1	12.3		
	1	Count	43	51		
		% within gender	26.5	31.5		
	2	Count	57	48		
		% within gender	35.2	29.6		
	3	Count	35	32		
		% within gender	21.6	19.8		
	4	Count	9	11		
		% within gender	5.6	6.8		

Smile line	Low	Count	39	47	16.908	0.000^*∗∗*^
		% within gender	24.1	29.0		
	Medium	Count	83	48		
		% within gender	51.2	29.6		
	High	Count	40	67		
		% within gender	24.7	41.4		

Parallelism	No	Count	51	122	62.523	0.000^*∗∗*^
		% within gender	31.5	75.3		
	Yes	Count	111	40		
		% within gender	68.5	24.7		

Buccal corridor	Absent	Count	106	97	1.068	0.301
		% within gender	65.4	59.9		
	Present	Count	56	65		
		% within gender	34.6	40.1		

^*∗∗*^ Significant at 0.01 level; ^*∗*^*P* < 0.05, statistically significant.

**Table 3:** Correlation matrix for the association between smile parameters.

		Midline shift	Lip line	Parallelism	Buccal corridor
Spearman's rho	Midline shift	1.000	0.021−	0.062−	0.139^*∗*^
	Lip line	—	1.000	0.059	0.027
	Parallelism	—	—	1.000	0.033
	Buccal corridor	—	—	—	1.000

^*∗*^Correlation is significant at the 0.05 level (2-tailed).

## Data Availability

Due to the ethical and legal responsibility to respect participants' rights to privacy and to protect their identity, the clinical dataset is not publicly available.
